# Tau Reduction Diminishes Spatial Learning and Memory Deficits after Mild Repetitive Traumatic Brain Injury in Mice

**DOI:** 10.1371/journal.pone.0115765

**Published:** 2014-12-31

**Authors:** Jason S. Cheng, Ryan Craft, Gui-Qiu Yu, Kaitlyn Ho, Xin Wang, Geetha Mohan, Sergey Mangnitsky, Ravikumar Ponnusamy, Lennart Mucke

**Affiliations:** 1 Gladstone Institute of Neurological Disease, San Francisco, California, United States of America; 2 Department of Neurological Surgery, University of California San Francisco, San Francisco, California, United States of America; 3 Department of Neurology, University of California San Francisco, California, United States of America; 4 Department of Radiology and Biomedical Imaging, University of California San Francisco, San Francisco, California, United States of America; Nathan Kline Institute and New York University Langone Medical Center, United States of America

## Abstract

**Objective:**

Because reduction of the microtubule-associated protein Tau has beneficial effects in mouse models of Alzheimer's disease and epilepsy, we wanted to determine whether this strategy can also improve the outcome of mild traumatic brain injury (TBI).

**Methods:**

We adapted a mild frontal impact model of TBI for wildtype C57Bl/6J mice and characterized the behavioral deficits it causes in these animals. The Barnes maze, Y maze, contextual and cued fear conditioning, elevated plus maze, open field, balance beam, and forced swim test were used to assess different behavioral functions. Magnetic resonance imaging (MRI, 7 Tesla) and histological analysis of brain sections were used to look for neuropathological alterations. We also compared the functional effects of this TBI model and of controlled cortical impact in mice with two, one or no *Tau* alleles.

**Results:**

Repeated (2-hit), but not single (1-hit), mild frontal impact impaired spatial learning and memory in wildtype mice as determined by testing of mice in the Barnes maze one month after the injury. Locomotor activity, anxiety, depression and fear related behaviors did not differ between injured and sham-injured mice. MRI imaging did not reveal focal injury or mass lesions shortly after the injury. Complete ablation or partial reduction of tau prevented deficits in spatial learning and memory after repeated mild frontal impact. Complete tau ablation also showed a trend towards protection after a single controlled cortical impact. Complete or partial reduction of tau also reduced the level of axonopathy in the corpus callosum after repeated mild frontal impact.

**Interpretation:**

Tau promotes or enables the development of learning and memory deficits and of axonopathy after mild TBI, and tau reduction counteracts these adverse effects.

## Introduction

Each year, 1.7 million Americans suffer mild traumatic brain injury (TBI) and half of them experience an acute loss of consciousness (LOC) as a result of the injury [1]. The diagnostic criteria for mild TBI include ≤30 min LOC, <24 h of confusion or memory loss, and normal brain imaging [2]. The etiologies of these injuries range from minor traffic accidents and sports-related concussions to falls and mild blast injuries in military personnel [2]. Many cases of mild TBI, especially in the civilian setting, do not reach medical attention and almost all recover from the acute effects of the TBI without treatment. However, studies examining the neurocognitive performance of these patients for up to one year after the injury have revealed deficits in visuospatial learning, executive function, and working memory [3]–[5]. Even more disconcerting are the results of long-term studies, showing an increased risk of developing Alzheimer's disease (AD) or Parkinson's disease even after a single mild TBI, as compared to uninjured age-matched controls [6]–[9]. Thus, the delayed effects of mild TBI can be disabling and represent an important public health problem [10], [11].

Histopathological studies of *postmortem* brain tissues from professional athletes who experienced repeated concussions during their careers identified the presence of neurofibrillary tangles (NFT) and neuropil threads, pathological hallmarks of chronic traumatic encephalopathy [6], [12]. These inclusions consist primarily of phosphorylated forms of the microtubule-associated protein Tau and are similar in appearance to those found in AD [12]. While the role of Tau in mild TBI remains to be elucidated, endogenous wildtype Tau appears to be required for amyloid-beta (Aβ) peptides and apolipoprotein (apo) E4 to cause synaptic, network and cognitive deficits in mouse models of AD [13]–[16]. Tau reduction has also been shown to block epileptogenesis of diverse causes, including epileptic activity triggered by pharmacological blockade of GABA_A_ channels [14], [17], genetic ablation of the voltage-gated potassium channel subunit K_v_1.1 [18], depletion of ethanolamine kinase or of the K^+^–Cl^−^ cotransporter [18], or depletion of the voltage-gated sodium channel subunit Na_v_1.1 [19]. The mechanisms underlying these beneficial effects of Tau reduction remain to be determined, but may include alterations in neuronal excitability, synaptic scaffolding, and neurogenesis (see [20] for review).

Recent evidence suggests that mild TBI may share pathogenic mechanisms with AD, including aberrant network excitability, cytoskeletal disruption, and inflammation [21]–[23]. Taken together, these findings raise the question of whether Tau reduction can also protect the brain against the long-term sequelae of mild TBI. To address this question, we adapted a rat model of mild, repetitive TBI [24] for mice. We demonstrate that this mouse TBI model results in early post-traumatic deficits in spatial learning and memory and causes long-term neurodegenerative changes. We further show that genetic reduction of Tau is able to diminish these abnormalities.

## Materials and Methods

### Mice

We used male and female C57Bl/6J mice at 4–6 months of age. Mice were group housed on a regular 12-h light/dark cycle. Food (PicoLab Rodent Diet 20,5053) and water were provided *ad libitum*. *Tau^–/–^* mice [25] were obtained from Jackson Laboratories, strain #007251. *Tau^+/–^* mice were generated by breeding *Tau^+/+^* mice (C57Bl/6J strain, The Jackson Laboratory) with *Tau^–/–^* mice of the same strain. Littermates of three genotypes (*Tau^+/+^*, *Tau^+/–^*, *Tau^–/–^*) were generated by breeding *Tau^+/–^* mice with *Tau^+/–^* littermates.

### Ethics Statement

This study was carried out in strict accordance with the recommendations in the Guide for the Care and Use of Laboratory Animals of the National Institutes of Health. All procedures were approved by the Animal Care and Use Committee of the University of California, San Francisco and all efforts were made to minimize suffering.

### Injury Models

Mice were randomly assigned to undergo TBI or sham surgery. Three types of mild TBI were used: frontal impact (2-hit vs 1-hit) and controlled cortical impact (1-hit). After injury, mice were injected with buprenorphine (0.01 mg/kg SC) and checked hourly for 6 hours and then daily. They were allowed to recover for a minimum of 2 weeks before the initiation of behavioral testing.

### Frontal Impact

We adapted a rat model of diffuse cortical injury [24] for mice as follows. A smooth plexi-glass platform (20 cm wide, 25 cm long and 5 cm deep) was placed at the base of a steel ramp (112 cm long with highest point 76 cm above table) and used as a support platform for the anesthetized mice. A coupling device was constructed from a polycarbonate cylinder (2.5 cm in diameter and 3.8 cm long) and had two prongs (3 mm flattened steel nails set 6 mm apart) in the center. Mice were anesthetized with isoflurane (3% at 6 liters per min) before and during the injury. After 2.5 min of anesthesia induction, they were placed on the edge of the platform and the prongs of the coupling device were placed against their frontal zygomatic processes bilaterally. A 56-gram steel ball was then released from a vertical height of 71 cm on the ramp, which impacted the coupling device and transduced the kinetic energy into the skull base of the mouse. Sham animals underwent the same anesthetic treatment and were placed on the support platform with engagement of the coupling device, but did not receive a frontal impact injury.

### Controlled Cortical Impact (CCI)

Mice were induced for 2 min with 3% isoflurane and maintained under this anesthesia using a nose cone for the duration of the procedure. Bupivacaine (8 mg/kg SC) was administered to the scalp. After shaving, a linear incision was made in the midline followed by a 2.5 mm circular craniotomy. The CCI device (Hatteras Instruments, Caray, NC) was attached to a stereotactic frame and positioned 1.5 mm lateral and 2.3 mm posterior to Bregma. Injury was inflicted using a 1.5-mm circular, flat impactor tip traveling at a speed of 3 m/s and penetrating to a depth of 1.5 mm for 150 ms. After injury, the craniotomy bone was replaced and the scalp closed using dermabond cement. Sham-injured animals underwent anesthesia and craniotomy but not cortical impact.

### Behavioral Experiments

Mouse cohorts underwent a maximum of three of the behavioral tests described below. In instances where more than one test was performed on the same group of mice, the order of testing was selected to minimize test interactions. Testing in the open field and elevated plus maze preceded testing in the Barnes maze, and testing in the Y-maze and on the balance beam preceded contextual and cued fear conditioning. The forced swim test was carried out in a mouse cohort that did not undergo other behavioral tests.

#### Barnes Maze

The maze consisted of a circular platform (91.4 cm diameter) with 20 holes around the periphery (5.1 cm diameter) with an escape box attached to the bottom of one of the holes and shallow boxes attached to the bottom of the other holes. The lights were kept bright (650 lux) to motivate mice to find and enter the escape box. Visual extra-maze cues were present on 3 walls of the room at a 1.5–1.8 m distance from the maze. For all trials, mice were placed individually in a cylindrical black start chamber in the center of the maze for 10 s, which was then lifted to start the test. During an adaptation period, mice were guided to the escape tunnel and allowed to stay there for 2 min. During a spatial acquisition period, a total of 10 acquisition trials (2 trials per day with an inter-trial interval of 15 min) were performed; mice were allowed to explore the maze freely for 3 min. Each trial ended when the mouse entered the escape tunnel or after 3 min had elapsed. Mice that did not find the tunnel were guided to it. All mice were allowed to remain in the tunnel for 1 min. During the probe trial conducted 1 day after the last training trial, the escape tunnel was replaced by a shallow box and mice were allowed to explore the maze for 90 s. Mice were video recorded and the time (“latency”) and path length (“distance”) taken to the target location during the probe trials were measured. For mice that did not reach the target location, total testing time (90 s) and total distance moved were used for analysis in lieu of latency and distance to target.

#### Contextual and Cued Fear Conditioning

Mice were tested in a 3-day paradigm as described [26]. Briefly, on the first day, mice were placed into a novel context (Med Associates, Inc., St. Albans, VT) in which they underwent 3 training trials, each consisting of an auditory cue (2800 Hz, 85 dB, 30 s) that co-terminated with a 2-s foot shock (0.45 mA) during the 29^th^ and 30^th^ seconds. The inter-trial interval was 2 min. On the second day, animals were placed in the same context and monitored for freezing behavior for 8 min. On the third day, mice were placed in a different context and exposed to the same 3 auditory cues as on day 1 but without receiving a foot shock. The percent of time mice spent freezing was recorded before and after the auditory cues.

#### Elevated Plus Maze

Mice were tested for a total of 10 min in a dimly lit room. The test was initiated by placing mice at the intersection between the open and closed arms. Basic locomotor activity and percent of time spent in open versus closed arms were recorded as described [27].

#### Open Field

Mice were tested for total movements and rearings as described [27].

#### Balance Beam

Mice were trained to traverse a square beam measuring 6 mm×6 mm×61 cm and tested using a square beam measuring 3 mm×3 mm×61 cm. The total number of foot slips and latency to cross the beam were recorded as described [28].

#### Y-Maze

The test was initiated by placing mice into one arm of the Y-maze. Total movements and the number and percent of alternations were recorded for 6 min and analyzed as described [27].

#### Forced Swim Test

Mice were placed in a clear polycarbonate cylinder measuring 31 cm in diameter and 76 cm in height filled to 48 cm with room-temperature tap water. Time to immobilization after immersion was recorded up to a 6 min maximum as described [29].

#### 7T MRI Imaging

Mice were anesthetized with isoflurane while MRI data were acquired with a 7T MRI scanner (Agilent/Varian, Santa Clara, CA) using a 3D gradient echo sequence to produce T1 and T2* weighted images as described [30].

### Immunohistochemistry

Twelve months after 2-hit injury, mice were anesthetized with Avertin (tribromomethanol, 250 mg/kg) and perfused transcardially with 0.9% saline for 1 min. Brains were removed and post-fixed in 4% paraformaldehyde (PFA) at 4°C for 24 hrs, followed by incubation in 30% sucrose for 1–3 days at 4°C. Hemi-brains were then sectioned coronally to a thickness of 30 µm using a freezing microtome (Leica SM 2000R). Sections were stained with the following antibodies: mAPP (Millipore, 22C11, dilution 1∶5000), GFAP (Millipore, MAB360 Clone GA5, dilution 1∶2000), and Iba-1 (Wako Chemicals, 019-19741, 1∶1000). An avidin-biotin complex kit (Vector Laboratories) and 3,3′-diaminobenzidine tetrahydrochloride (Vector Laboratories) were used to visualize antibody labeling. Silver staining was performed using the Bielschowsky method as described [31]. Sections were imaged with a BZ-9000 automated microscope system (Keyence) using a 10X objective (Nikon).

Levels of axonopathy were determined by counting mAPP-positive profiles throughout the body of the corpus callosum in four sections per mouse. To detect astrocytosis and microgliosis, three non-overlapping areas (100 µm^2^ each) in the body of the corpus callosum were randomly selected in four sections per mouse and the average percent area occupied by GFAP or Iba-1 immunoreactivity was determined using ImageJ software (NIH), as described [32]. The average thickness of the corpus callosum was calculated by measuring its dorsoventral extent along the midline in four silver-stained coronal sections that were equally spaced along the anterior to posterior extent of the body of the corpus callosum, as described [32].

### Statistics

Statistical analyses were performed using SPSS 21 (IBM, Armonk, NY) and JMP (SAS, Cary, NC). Differences among multiple means were assessed by one-way or two-way ANOVA followed by post-hoc comparisons between groups by Tukey-Kramer test. Differences between two means were assessed with the Student's t-test. Learning curves in the Barnes maze were assessed with a linear mixed effects model and fitted using the SPSS package MIXED. The model included the following effects: Day, Injury, Tau, Injury*Tau, Day*Injury, Day*Tau, Day*Injury*Tau. A fixed effect for observations from trials 2, 4, 6, 8, and 10 was also included to allow for improvements from the first to the second trial on each day. Random mouse level intercepts and slopes accounted for the correlation among repeated observations. Significance was defined as p<0.05.

## Results

### Spatial learning and memory deficits one month after 2-hit, but not 1-hit, frontal injury

In humans, most concussive injuries occur in the anterior-posterior axis of the brain, a process that was not adequately simulated by previously available mouse models. In contrast, the Maryland TBI model for rats does simulate an angular, frontal impact and produces robust behavioral deficits [24]. We therefore adapted this approach for 4–6-month-old C57BL/6J mice. Wildtype mice were anesthetized with isoflurane and subjected to one frontal hit (1-hit), two frontal hits 48 hours apart (2-hit), or sham injury.

**Figure 1:**
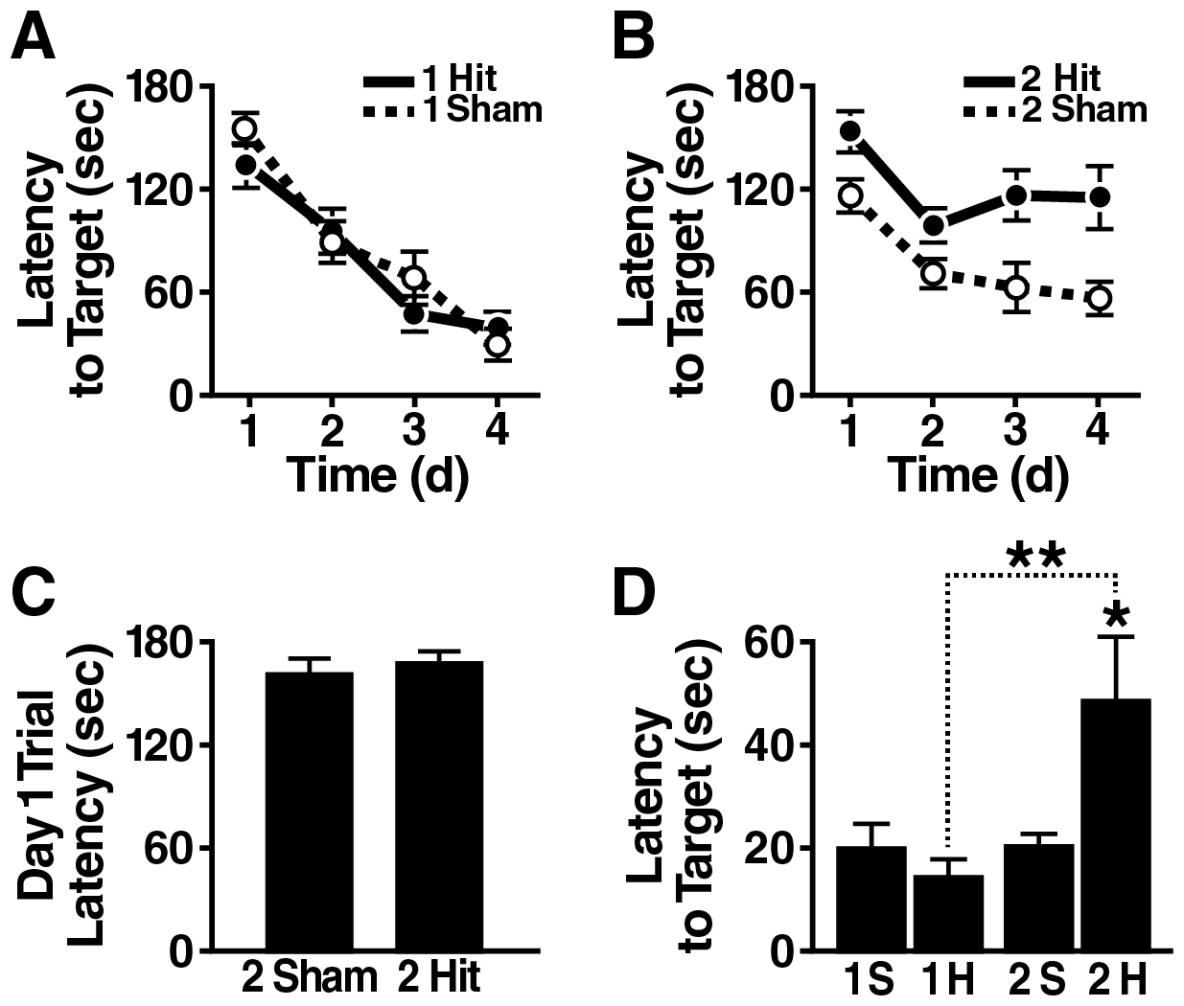
Wildtype mice show learning and memory deficits one month after 2-hit frontal injury. Wildtype mice (n = 9–10 per group) received a 1-hit or a 2-hit frontal impact injury or sham treatments, and were tested in the Barnes maze one month later. (A, B) Learning curves of the 1-hit (A) and 2-hit (B) groups, reflecting the time it took mice to find the target, averaged from 2 trials per day. Only the 2-hit group differed significantly from sham-treated controls (p = 0.0062 by linear mixed effects model analysis). (C) The 2-hit group and sham-treated controls showed a comparable latency to target during the first trial on the first training day. (D) Probe trial administered 24 h after the last training trial. *p<0.05, **p<0.01 vs corresponding sham group or as indicated by bracket. Sh, Sham; H, Hit. Data are means ± SEM.

Spatial learning and memory were assessed four weeks after the injury with the Barnes maze test. During the acquisition phase of this test, 1-hit mice learned the task as well as controls (Fig. 1A). In contrast, 2-hit mice showed significant impairments in task acquisition (Fig. 1B). These impairments likely reflect a learning deficit rather than nonspecific performance problems as 2-hit mice and controls performed similarly during the first trial on the first day of training (Fig. 1C).

Learning and memory retention were further assessed in a probe trial administered 24 hours after the last training trial. Whereas 1-hit mice performed at control levels, 2-hit mice were impaired, taking significantly longer to reach the target hole than controls and 1-hit mice (Fig. 1D). Similar findings were obtained in two replicate cohorts of mice (S1A–D Fig.).

### Two hit frontal injury does not impair several other behavioral functions

**Figure 2:**
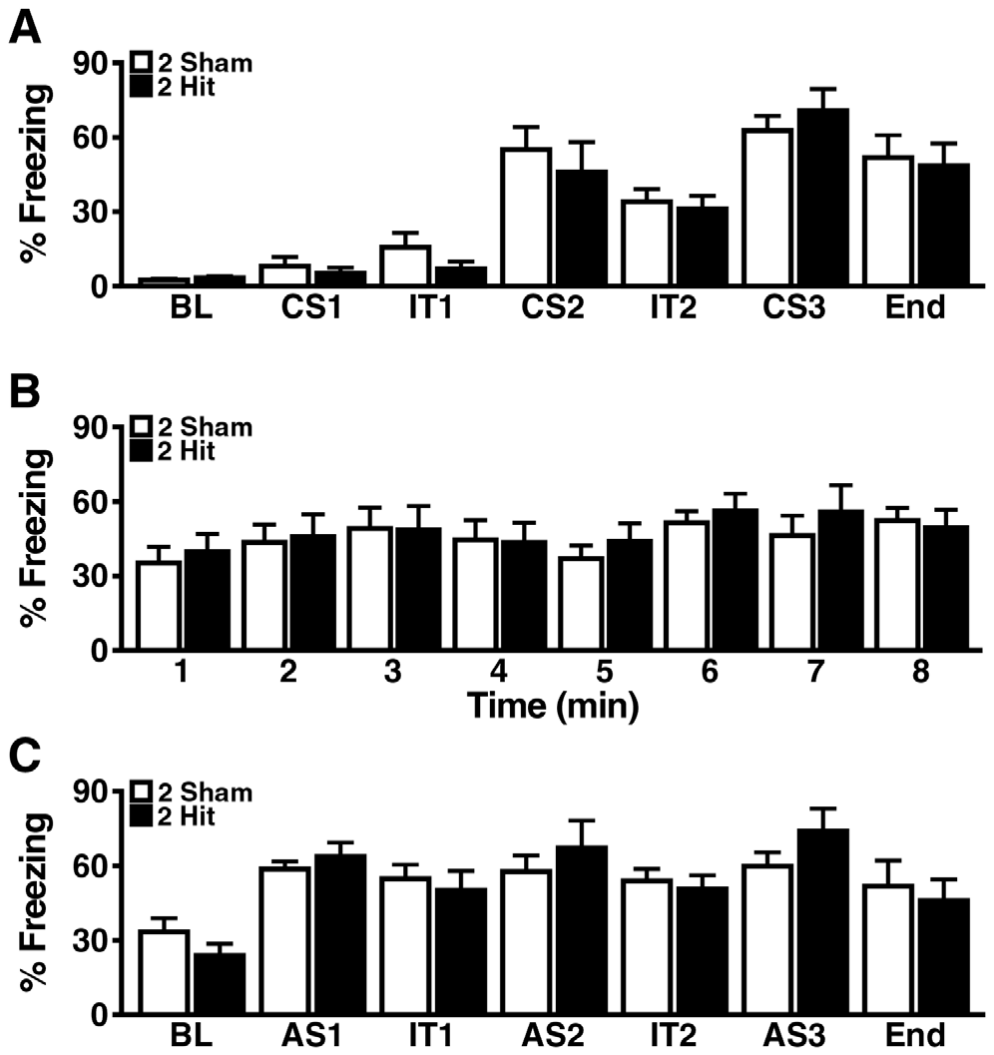
Wildtype mice show no impairments in context or cued fear learning and memory after 2-hit injury. Wildtype mice (n = 8–10 per group) received a 2-hit frontal impact injury or sham treatment, and underwent cued and contextual fear conditioning 1 month later. (A) On day 1, the sham-treated and 2-hit groups showed comparable amounts of freezing at baseline as well as during and between training trials. BL, baseline (3 min); CS, conditional stimulus (auditory stimulus followed by foot shock); IT, interval between CS (2 min); End, period following last CS (2 min). See methods for additional details. (B, C) Both groups also showed a comparable amount of freezing when they were introduced into the same context on day 2 without receiving an auditory stimulus or foot shock (B) or into a novel environment on day 3 after hearing the auditory stimulus without receiving a foot shock (C). AS, auditory stimulus. One-way ANOVA revealed no significant differences between the groups (A–C). Data are means ± SEM.

**Figure 3:**
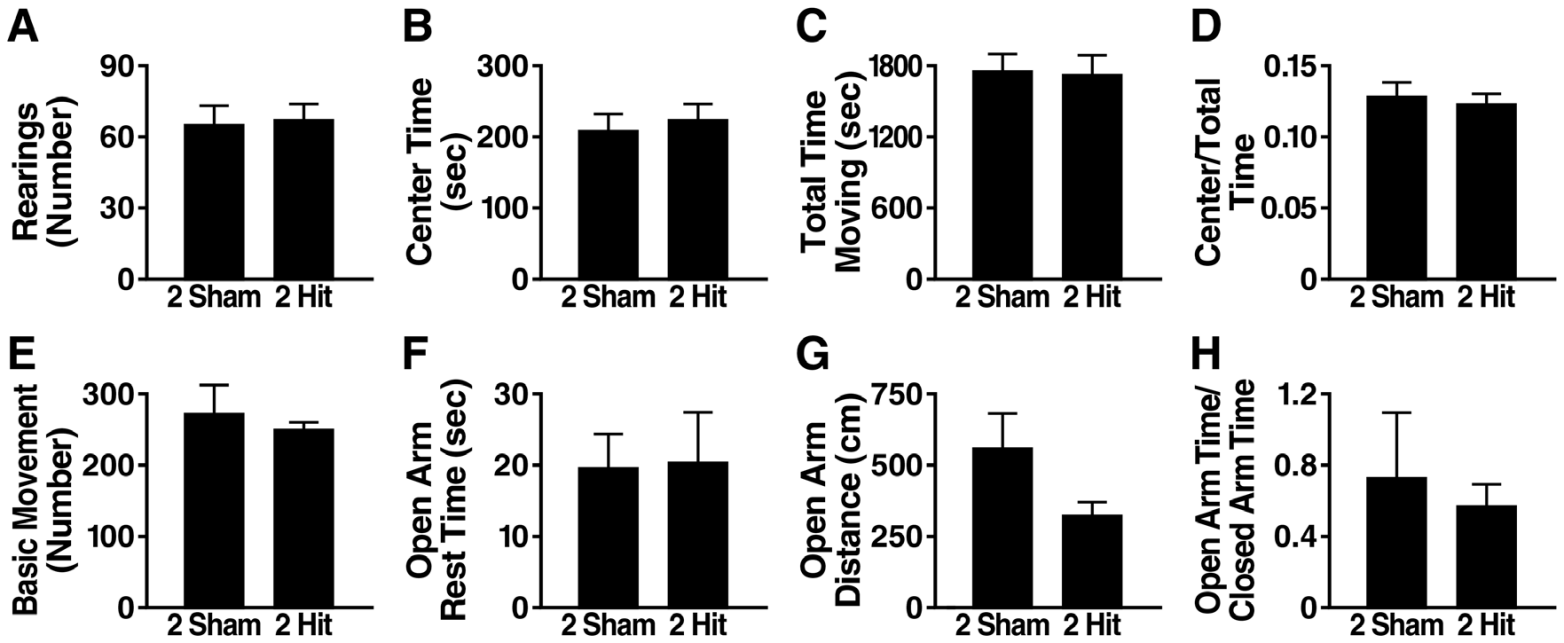
Wildtype mice show no alterations in exploratory activity or anxiety-like behavior after 2-hit injury. Wildtype mice (n = 8–10 per group) received a 2-hit frontal impact injury or sham treatment, followed by assessment in different behavioral tests. (A–D) Open field activity 2 weeks post-injury. (E–H) Behavior in elevated plus maze 4 weeks post-injury. Student's t test revealed no significant differences between the 2-hit and sham treated groups for any of the tests and outcome measures. Data are means ± SEM.

**Figure 4:**
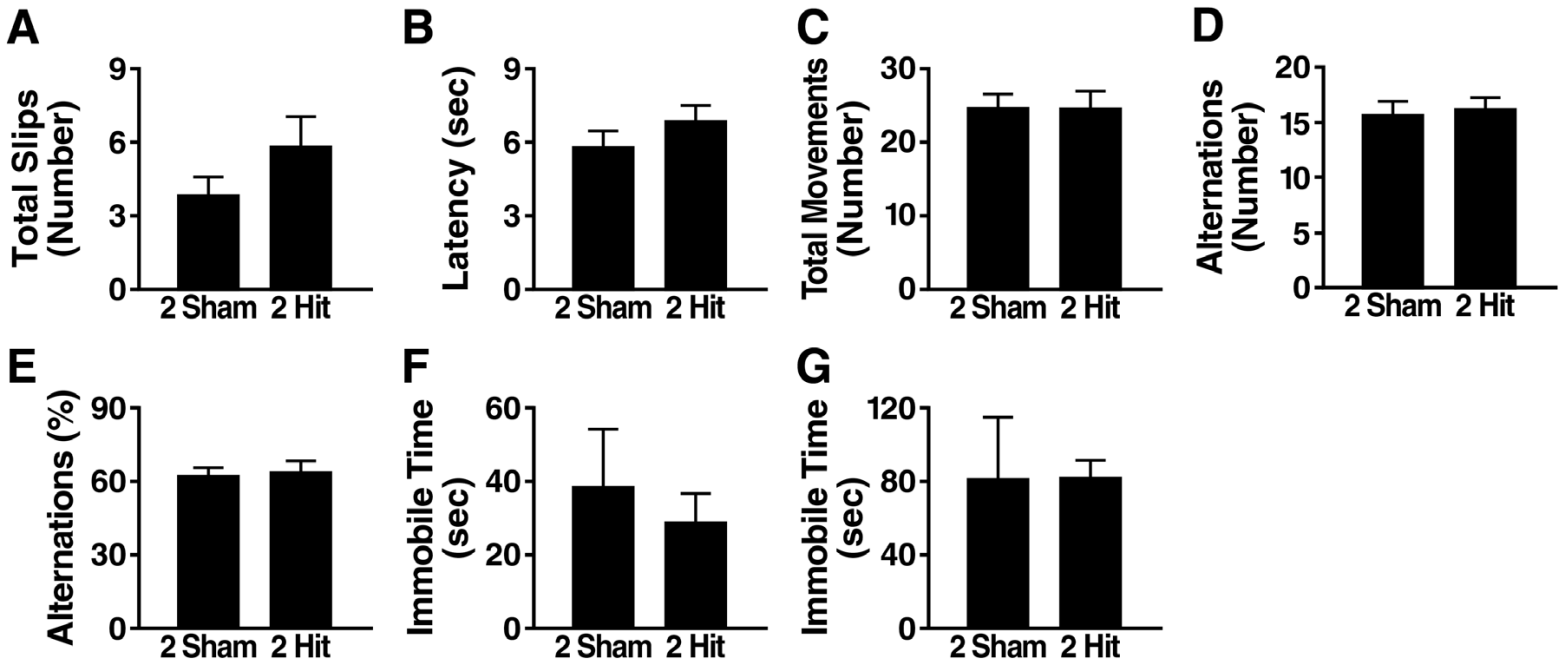
Wildtype mice show no alterations in motor performance or depression-like behavior after 2-hit injury. Wildtype mice (n = 8–10 per group) received a 2-hit frontal impact injury or sham treatment, followed by behavioral assessment. (A–B) Balance beam performance 3 weeks post-injury. (C–E) Y-maze activity 2 weeks post-injury. (F–G) Forced swim test 5 days (F) and 6 months (G) post-injury. Student's t test revealed no significant differences between the 2-hit and sham treated groups for any of the tests and outcome measures. Data are means ± SEM.

To compare their fear responses and associative learning and memory, we tested 2-hit mice and controls in a 3-day fear conditioning paradigm 4 weeks after injury. Similar results were obtained in both groups of mice in regards to all outcome measures examined (Fig. 2A–C). We also did not detect significant differences between 2-hit mice and controls 2–4 weeks after injury in the open field (Fig. 3A–D), a test of locomotor activity and anxiety [33], in the elevated plus maze (Fig. 3E–H), a test of exploratory activity and anxiety [33], on the balance beam (Fig. 4A–B), a test of motor coordination (23), and in the Y-maze (Fig. 4C–E), a test of exploratory activity and working memory (22). Depression-like behavior was assessed with the forced swim test at 5 days and 6 months post-injury. No differences in immobile time were detected between 2-hit mice and controls at either time point (Fig. 4F–G).

### Two hit frontal injury does not cause radiological abnormalities detectable by 7T MRI

**Figure 5:**
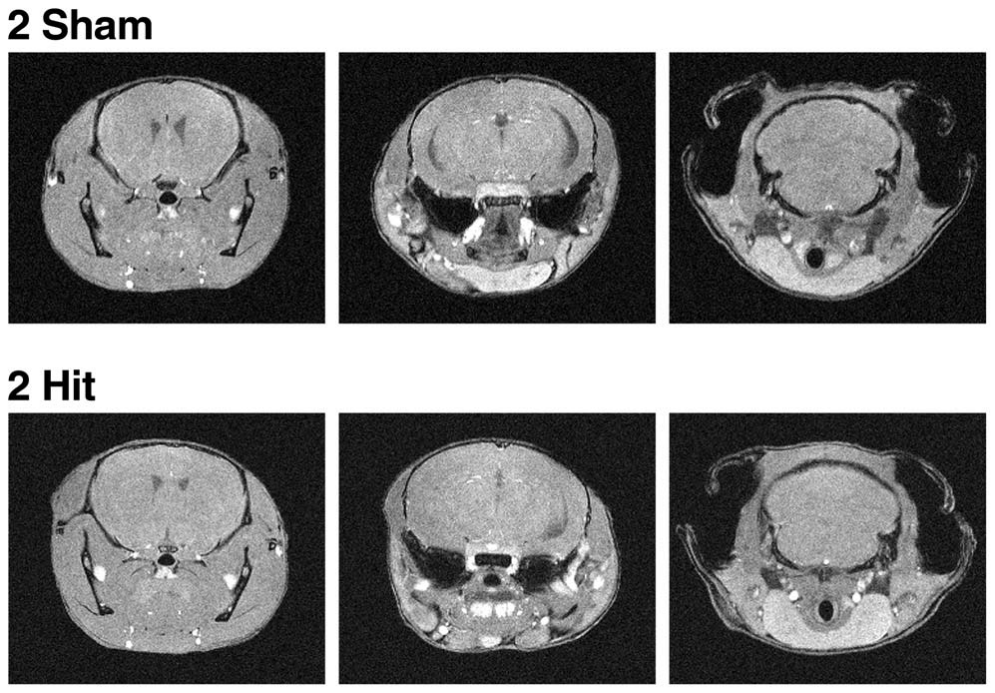
Wildtype mice show no focal radiographic abnormalities on MRI after 2-hit injury. Wildtype mice (n = 6 per group) received a 2-hit frontal impact injury or sham treatment. Their brains were imaged by MRI two days after the second injury. (A–B) Representative T1 weighted coronal images of sham treated (A) and 2-hit injured (B) mice.

Some 2-hit mice and controls (n = 6 per group) were subjected to brain imaging by 7T MRI 2 days after the second injury. This analysis revealed no evidence for contusions, hemorrhage, ischemia, or hippocampal injury in 2-hit mice (Fig. 5 and data not shown).

### Tau reduction diminishes spatial learning and memory deficits after 2-hit frontal injury

**Figure 6:**
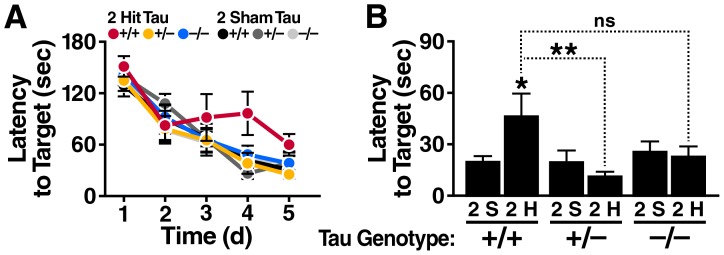
Tau reduction diminishes learning and memory deficits caused by 2-hit frontal injury. *Tau* ^+/+^, *Tau* ^+/–^ and *Tau* ^–/–^ mice (n = 9–10 per genotype and treatment) received a 2-hit frontal impact injury or sham treatment, and were tested in the Barnes maze one month later. (A) Learning curves of the indicated groups. By linear mixed effects model analysis, injured *Tau* ^+/+^ mice differed from sham treated *Tau* ^+/+^ mice (p = 0.0001), injured *Tau* ^+/–^ mice (p = 0.02) and possibly also injured *Tau* ^–/–^ mice (p = 0.09). Injured *Tau* ^+/–^ and *Tau* ^–/–^ mice did not differ from each other or from their respective sham treated controls. (B) Probe trial administered 24 h after the last training trial. Two-way ANOVA revealed a *Tau* genotype effect (p = 0.016, F(2, 53) = 4.54) and an interaction between *Tau* genotype and injury (p = 0.011, F(2, 53) = 4.95). *p<0.05, **p<0.01 vs. sham treated group of same genotype or as indicated by brackets (Tukey-Kramer test). ns, not significant; Sh, Sham. Data are means ± SEM.

To assess the effects of complete and partial Tau reduction on functional deficits caused by 2-hit frontal impact, we subjected 4–6-month-old *Tau* ^+/+^, *Tau* ^+/–^ and *Tau* ^–/–^ mice to this injury and compared their spatial learning and memory in the Barnes maze 4 weeks later. Two hit mice showed significant impairments in learning this task relative to sham-injured controls only on the *Tau* ^+/+^, but not on the *Tau* ^–/–^ or *Tau* ^+/–^, background (Fig. 6A).

Similar results were obtained in a probe test administered 24 hours after the last training trial, with 2-hit mice showing significant impairments relative to sham-injured controls on the *Tau* ^+/+^, but not the *Tau* ^–/–^ or *Tau* ^+/–^, background (Fig. 6B). Thus, even partial tau reduction is sufficient to significantly reduce spatial learning and memory deficits caused by 2-hit frontal injury. The protective effect of partial Tau reduction was confirmed in an independent cohort of mice (S1C, D Fig.).

### Tau reduction is less efficacious in a model of focal cortical injury

**Figure 7:**
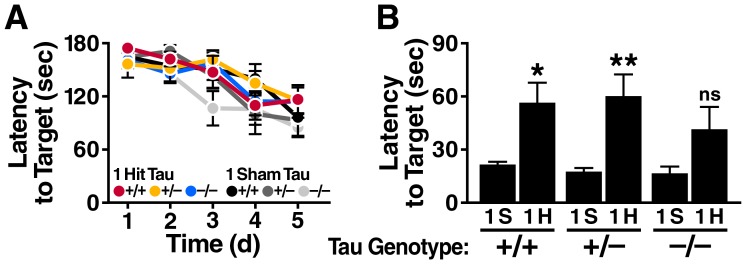
Tau reduction has less effect on memory deficits caused by controlled cortical impact (CCI) injury. *Tau* ^+/+^, *Tau* ^+/–^ and *Tau* ^–/–^ mice (n = 7–10 per genotype and treatment) received a single focal CCI injury to the right frontal cortex or a sham treatment, and were tested in the Barnes maze one month later. (A) Learning curves did not differ significantly among the indicated groups (linear mixed effects model analysis). (B) Probe trial administered 24 h after the last training trial. Two-way ANOVA revealed an injury effect (p<0.0001, F(1, 46) = 29.15) but no *Tau* genotype effect or interaction between injury and *Tau* genotype. *p<0.05, **p<0.01 vs. sham treated group of same genotype. ns, not significant. Sh, Sham. Data are means ± SEM.

To determine whether Tau reduction is also beneficial in another model of TBI, we subjected an independent cohort of 4–6-month-old *Tau* ^+/+^, *Tau* ^+/–^ and *Tau* ^–/–^ mice to a single unilateral controlled cortical impact (CCI), a model of focal and secondarily diffuse brain injury [34]. Mice were tested in the Barnes maze one month after the injury. No significant differences in task acquisition were identified among the groups (Fig. 7A). However, the 24-hour probe trial revealed significant deficits in injured mice relative to sham-injured controls on the *Tau* ^+/+^ and *Tau* ^+/–^ backgrounds (Fig. 7B). Although injured mice also showed a trend towards impairment on the *Tau* ^–/–^ background (Fig. 7B), this trend did not reach statistical significance, suggesting relative protection by the complete ablation of Tau.

### Tau reduction prevents chronic axonopathy caused by 2-hit frontal injury

**Figure 8:**
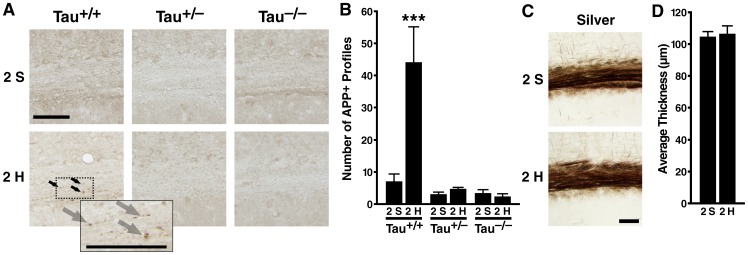
Tau reduction prevents chronic axonopathy in corpus callosum of 2-hit mice. *Tau* ^+/+^, *Tau* ^+/–^ and *Tau* ^–/–^ mice (n = 8–10 per genotype and treatment) received a 2-hit frontal impact injury or sham treatment, underwent behavioral testing 2–6 weeks later, and were analyzed histologically 12 months after the initial injury. (A, B) Coronal brain sections were immunostained for mAPP (22C11). (A) Photomicrographs depicting abnormal mAPP-positive profiles in the corpus callosum of a 2-hit *Tau* ^+/+^ mouse that are not seen in the other mice. (B) Quantitation of mAPP-positive profiles in the corpus callosum. Two-way ANOVA revealed a *Tau* genotype effect (p = 0.0011, F (2, 45) = 7.94), a 2-hit injury effect (p = 0.027, F (1, 45) = 5.25) and an interaction between *Tau* genotype and injury (p = 0.008, F (2, 45) = 5.42). ***p<0.001 vs. sham-treated group of same genotype (Tukey-Kramer test). (C,D) Axons in the corpus callosum of 2-hit and 2-sham *Tau* ^+/+^ mice were labeled by silver staining. (C) Representative photomicrographs. (D) The thickness of the corpus callosum was quantitated as described in Methods. Student's t test revealed no significant difference between the groups. Sh, Sham, H, Hit. Scale bar: 80 µm. Data are means ± SEM.

To assess whether 2-hit frontal injury leads to long term neurodegenerative changes, we analyzed a cohort of *Tau* ^+/+^, *Tau* ^+/–^ and *Tau* ^–/–^ mice immunohistochemically 12 months after 2-hit frontal impact or 2-sham treatment. The extent of axonal injury was quantified by counting mAPP-positive profiles in the body of the corpus callosum as described [32]. On the *Tau* ^+/+^ background, but not on the *Tau* ^+/–^ or *Tau* ^–/–^ background, 2-hit mice showed a marked increase in the number of mAPP-positive profiles compared to 2-sham controls (Fig. 8A, B). Indeed, 2-hit frontal injury did not increase callosal levels of mAPP-positive profiles above control levels in mice whose tau expression was eliminated or reduced by 50% (Fig. 8B). This injury did not affect the thickness of the corpus callosum even in *Tau* ^+/+^ mice (Fig. 8C and D).

### No evidence for prolonged gliosis in wildtype mice after 2-hit frontal injury

**Figure 9:**
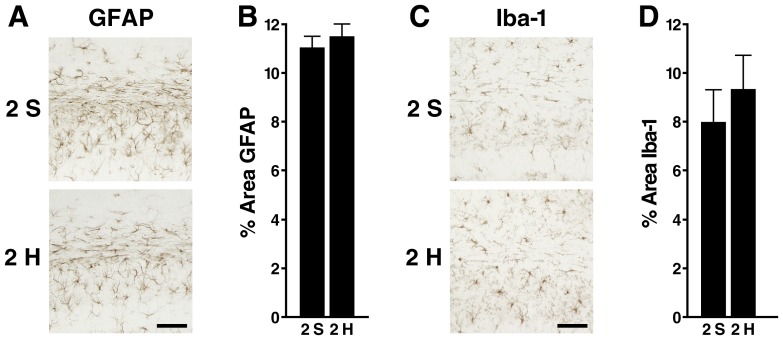
Wildtype mice have no callosal astrocytosis or microgliosis 12 months after 2-hit frontal injury. Wildtype mice (n = 8–10 per group) received a 2-hit frontal impact injury or sham treatment, underwent behavioral testing 2–6 weeks later, and were analyzed histologically 12 months after the initial injury. Coronal brain sections were immunostained for GFAP or Iba-1(A, B) Representative images (A) and quantitation (B) of GFAP immunoreactivity in the corpus callosum. (C, D) Representative images (C) and quantitation (D) of Iba-1 immunoreactivity in the corpus callosum. Sh, Sham, H, Hit. Scale bar: 80 µm. Data are means ± SEM.

To determine whether 2-hit frontal injury causes prolonged astrocytosis or microgliosis, we obtained brain sections from wildtype mice 12 months after injury and immunostained them for the astroglial marker GFAP or the microglial marker Iba-1. Two-hit and 2-sham mice showed similar levels of GFAP and Iba-1 immunoreactivity in the corpus callosum (Fig. 9A–D).

## Discussion

Our study demonstrates that partial reduction of endogenous tau can protect against spatial learning and memory deficits and chronic axonopathy caused by mild TBI. These findings were obtained in a new mouse model of mild, repetitive frontal impact injury that we developed based on a rat model reported by Kilbourne et al. [24]. In this mouse model, 2-hit, but not 1-hit, injury causes spatial, but not associative, learning and memory deficits, which were detectable one month after the injury. No potentially confounding deficits were observed in depression-related behavior, exploratory activity, anxiety, and motor functions 2–4 weeks after injury.

Our impact model has several advantages over existing mouse models of mild TBI. First, it generates a frontal impact that does not cause radiological abnormalities on MRI. Second, it eliminates complications that can be associated with skin incision and craniotomy, including infection, unintended cerebral injury, and postoperative wound pain. Third, preservation of the calvarium and overlying soft tissue also increases the options for employing diagnostic and therapeutic interventions such as electroencephalographic (EEG) monitoring, placements of drug infusion pumps, and radiological imaging.

During injury, the vector of force occurs along the anterior-posterior plane of the head and parallel to the skull base. This directionality reproduces common human injury mechanisms occurring during falls, motor vehicle accidents, and sports- or combat-related concussions. Unlike other models that impact the skull near the vertex, the new model does not compress the brain against the skull base, minimizing collateral injury to the brainstem and cerebellum that can cause confounding deficits in survival, coordination and balance. Like many forms of human TBI, it primarily impacts frontal brain structures and creates brain shifts as well as linear and angular shearing forces that place long, white matter tracts at risk [35]. In contrast, CCI causes primarily a focal grey matter contusion, hematoma formation, and central necrosis that vary with the severity of the impact. Importantly, single impact CCI tends to spare the white matter tracts [35].

It is interesting in this regard that reducing tau, which can be detected in dendritic structures but is located primarily within axons [20], [36], [37], diminished spatial learning and memory deficits after mild, repetitive frontal impact, but not after CCI. Because tau reduction prevents Aβ-induced axonal transport deficits [38], it is tempting to speculate that it may also prevent axonal transport deficits caused by frontal impact. In addition, several lines of evidence suggest that tau reduction may prevent excitotoxin-induced abnormalities in neuronal activity [14], [17], [18], [36], which are associated with TBI [39], [40] and could disrupt specific cognitive functions.

Neuropathologically, our 2-hit model caused prolonged axonopathy, detectable in the corpus callosum of wildtype mice 12 months after injury, consistent with previous studies demonstrating neurodegenerative alterations after mild TBI in mice [32]. Notably, complete or partial reduction of tau effectively prevented this structural neuronal damage.

From a therapeutic perspective, it is encouraging that even partial reduction of tau was able to prevent spatial learning and memory deficits and neurodegenerative changes after mild, repetitive frontal impact injury, particularly in light of the recent demonstration that acute cerebral tau reduction in wildtype mice with antisense oligonucleotides is both feasible and well tolerated [17]. Even life-long partial tau reduction does not appear to be associated with any adverse effects in *Tau* ^+/–^ mice [41]. The extent to which complete genetic ablation of tau is well tolerated remains a matter of some controversy. Some groups, including our own, have demonstrated age-appropriate cognition and only subtle dopamine-independent motor deficits in old *Tau* ^–/–^ mice [42], whereas others have reported that such mice develop a parkinsonian phenotype and cognitive impairments [43]. To further assess the potential value of tau-reducing strategies for TBI, studies are needed to determine whether reduction of tau is also protective when implemented in adult animals before or after the injury has occurred.

## Supporting Information

S1 Fig **Independent experiments confirming learning and memory deficits of wildtype mice in the Barnes maze test one month after 2-hit frontal impact injury and protective effects of partial Tau reduction.** *Tau* ^+/+^, *Tau* ^+/–^ and *Tau* ^–/–^ mice (n = 8–10 per genotype and treatment) received a 2-hit frontal impact injury or sham treatment, and were tested in the Barnes maze one month later. (A, B) Learning curves (A) and 24-h probe trial (B) in wildtype mice. Linear mixed effects model analysis revealed a significant difference between the learning curves (p = 0.027). *p<0.05 by Student's t test. (C, D) Tau reduction effects on learning curves (C) and 24-h probe trial (D). Based on linear mixed effects model analysis of learning curves, injured *Tau* ^+/+^ mice differed from sham-treated *Tau* ^+/+^ mice (p<0.001) and injured *Tau* ^+/–^ mice (p <0.01), but not from injured *Tau* ^–/–^ mice (p = 0.15). Injured *Tau* ^+/–^ and *Tau* ^–/–^ mice did not differ from each other or from their respective sham-treated controls. Examination of probe trial results by two-way ANOVA revealed a *Tau* genotype effect (p = 0.022, F(2, 51) = 3.78) and an interaction between *Tau* genotype and injury (p = 0.02, F(2, 51) = 3.84). *p<0.05 vs. sham-treated group of same genotype or as indicated by brackets (Tukey-Kramer test). ns, not significant; Sh, Sham. Data are means ± SEM.
(TIF)
