# Supplementary figures and images for: Tau Reduction Diminishes Spatial Learning and Memory Deficits after Mild Repetitive Traumatic Brain Injury in Mice
Source: PLoS One. 2014 Dec 31;9(12):e115765. doi: 10.1371/journal.pone.0115765 (PMC4281043; doi:10.1371/journal.pone.0115765)

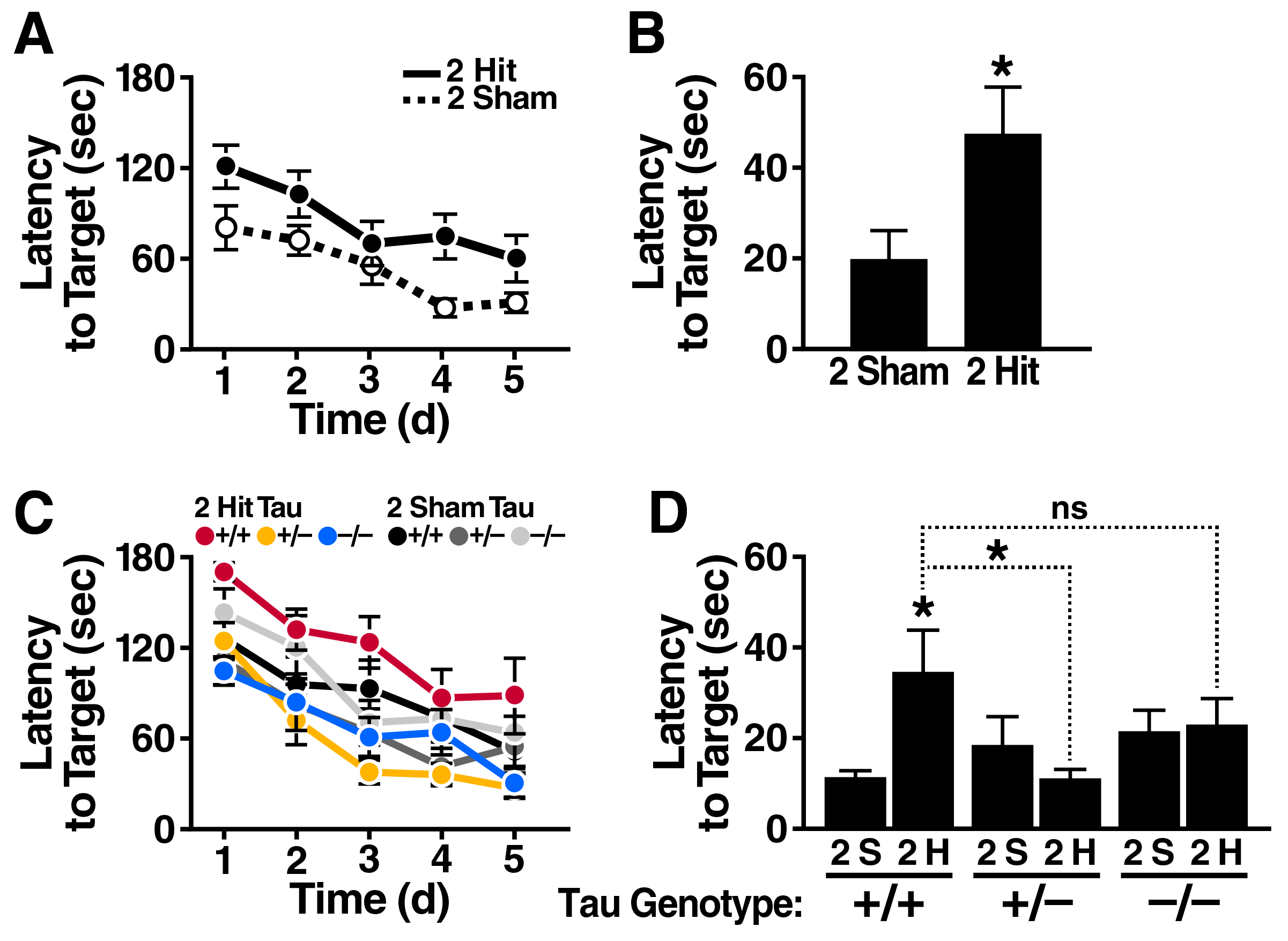

Supplement: S1 Fig — Independent experiments confirming learning and memory deficits of wildtype mice in the Barnes maze test one month after 2-hit frontal impact injury and protective effects of partial Tau reduction. Tau +/+, Tau +/– and Tau –/– mice (n = 8–10 per genotype and treatment) received a 2-hit frontal impact injury or sham treatment, and were tested in the Barnes maze one month later. (A, B) Learning curves (A) and 24-h probe trial (B) in wildtype mice. Linear mixed effects model analysis revealed a significant difference between the learning curves (p = 0.027). *p<0.05 by Student's t test. (C, D) Tau reduction effects on learning curves (C) and 24-h probe trial (D). Based on linear mixed effects model analysis of learning curves, injured Tau +/+ mice differed from sham-treated Tau +/+ mice (p<0.001) and injured Tau +/– mice (p <0.01), but not from injured Tau –/– mice (p = 0.15). Injured Tau +/– and Tau –/– mice did not differ from each other or from their respective sham-treated controls. Examination of probe trial results by two-way ANOVA revealed a Tau genotype effect (p = 0.022, F(2, 51) = 3.78) and an interaction between Tau genotype and injury (p = 0.02, F(2, 51) = 3.84). *p<0.05 vs. sham-treated group of same genotype or as indicated by brackets (Tukey-Kramer test). ns, not significant; Sh, Sham. Data are means ± SEM. (TIF) [file pone.0115765.s001.tif]
